# Antiphospholipid antibody production in pregnant women with SARS-CoV-2 infection and its impact on maternal and fetal outcomes

**DOI:** 10.3389/fimmu.2026.1810392

**Published:** 2026-04-10

**Authors:** Shenglong Ye, Yilin Wang, Jingjing Yang, Yang Zhang, Zhe Chen, Ran Hu, Chun Li, Xue Xu, Meiying Liang

**Affiliations:** 1 Department of Obstetrics and Gynecology, Peking University People’s Hospital, Beijing, China; 2 Department of Rheumatology and Immunology, Peking University People’s Hospital, Beijing, China

**Keywords:** antiphospholipid antibodies, placental function, pregnancy outcomes, pregnant women, severe acute respiratory syndrome coronavirus 2 (SARS-COV-2)

## Abstract

**Objective:**

To investigate the prevalence of antiphospholipid antibodies (aPLs) in pregnant women infected with severe acute respiratory syndrome coronavirus 2 (SARS-CoV-2) and their impact on maternal-fetal outcomes, with an emphasis on gestational stage-specific differences and lupus anticoagulant (LAC) positivity.

**Methods:**

This prospective, observational cohort study was conducted at the Department of Obstetrics, Peking University People’s Hospital, from December 2022 to January 2023. Eligible participants were singleton pregnant women without pre-existing pregnancy complications who tested positive for SARS-CoV-2. Serum samples were collected 2–4 weeks post-infection for testing of aPLs, including LAC, anti-β2-glycoprotein I (aβ2GPI), and anticardiolipin antibodies. Maternal and fetal outcomes were monitored according to standard prenatal care protocols, with specific attention to placental dysfunction related complications.

**Results:**

The overall prevalence of aPLs in pregnant women infected with SARS-CoV-2 was 18.75%. All positive cases were single-antibody positive, either for aβ2GPI or LAC, with no instances of multiple antibody co-positivity. The prevalence of aPLs was significantly correlated with the gestational stage at the time of infection, rising from 5.88% in the first trimester to 16.00% in the second trimester and 26.31% in the third trimester (*P<0.05*). Notably, all LAC-positive cases (constituting 8.25% of total infections) were exclusive to women infected during the third trimester. The group with aPLs positive status experienced a significantly higher incidence of placental dysfunction related complications, such as gestational hypertension, oligohydramnios, and fetal growth restriction, compared to the aPLs-negative group (60.00% vs. 29.23%, *P=0.036*). The LAC-positive subgroup exhibited an even higher incidence of complications (71.43%, *P=0.037*), with numerically higher complication rates observed in LAC-positive cases than other aPLs. Univariate logistic regression analysis revealed that aPLs positivity (*P=0.030*) and specifically LAC positivity (*P=0.041*) were independent risk factors for placental dysfunction.

**Conclusion:**

SARS-CoV-2 infection during pregnancy significantly increases the prevalence of aPLs, with a pattern that is dependent on the gestational stage. Positivity for aPLs, particularly LAC, is an independent risk factor for complications related to placental dysfunction. Our findings suggest that pregnant women infected with SARS-CoV-2, especially those in the third trimester, should undergo aPLs screening 2–4 weeks post-infection, along with enhanced monitoring for placental dysfunction, there should be vigilant surveillance of infection status, coagulation function, and appropriate obstetric management.

## 1 Introduction

The global pandemic caused by severe acute respiratory syndrome coronavirus 2 (SARS-CoV-2) has attracted widespread attention due to its severe harm to human health. Studies on non-pregnant populations infected with SARS-CoV-2 have found a transient elevation of autoantibodies such as antiphospholipid antibodies (aPLs) during the acute phase of infection (1–3). High titers of aPLs are associated with adverse prognoses, including severe pneumonia and mortality. In addition, patients with severe SARS-CoV-2 infection have an increased risk of thrombotic events due to pathological hypercoagulability (4–6). Moreover, aPLs induced by infection can also lead to thrombosis and thrombocytopenia (7, 8).

Currently, research on pregnant populations is limited. Most existing studies suggest that pregnant women infected with SARS-CoV-2 are mostly asymptomatic or have mild symptoms (9), and the risk of neonatal birth defects is not significantly increased (10). However, the incidence of maternal and fetal complications tends to rise (10, 11). Gestational complications include gestational hypertensive disorders, oligohydramnios, and fetal growth restriction (FGR) (12), which were categorized as placental dysfunction related disorders by some scholars (13). Some scholars propose that viral infection may cause placental dysfunction, but the specific mechanism by which the virus leads to adverse maternal and fetal outcomes remains unclear.

Previous studies have confirmed a clear correlation between aPLs and pathological pregnancy. aPLs can induce placental dysfunction related pathological pregnancies, such as recurrent miscarriage, early-onset preeclampsia, oligohydramnios, and FGR, through systemic inflammatory response, vascular endothelial injury, pathological hypercoagulability, and specific placental involvement in pregnant women. Epidemiological studies have shown that lupus anticoagulant (LAC), in particular, is an independent risk factor for adverse maternal and fetal outcomes (14). Existing studies have demonstrated that non pregnant individuals may develop transient aPLs positivity after SARS-CoV-2 infection, with an incidence ranging from 11% to 71% (15). This positivity is associated with the severity and prognosis of SARS-CoV-2 infection. However, there is only one published study on aPLs in pregnant women after SARS-CoV-2 infection (15). It reported a transient aPLs positivity rate of 11% in 151 pregnant inpatients with SARS-CoV-2 infection.

To further clarify the prevalence of aPLs in healthy pregnant women after SARS-CoV-2 infection and its impact on maternal and fetal outcomes, we conducted a single center clinical study during the period of normalized COVID-19 prevention and control in China, and the results are reported herein.

## 2 Materials and methods

### 2.1 Study population

This was a prospective cohort study involving singleton pregnant women who were infected with SARS-CoV-2 during pregnancy and met the inclusion criteria in the Department of Obstetrics of Peking University People’s Hospital from December 2022 to January 2023. All SARS-CoV-2 infections were caused by the Omicron variant, the dominant strain during the study period.

Inclusion criteria:

1. SARS-CoV-2 infection during pregnancy;
2. No preexisting pregnancy complications or comorbidities at the time of infection;
3. At least one serological aPLs test performed at 2–4 weeks post infection;
4. Complete maternal and fetal outcome data and case records available.

Exclusion criteria:

1. Complicated with autoimmune diseases, hematological diseases, hypertension, diabetes, nephropathy, or other chronic diseases;
2. Presence of pregnancy complications such as gestational hypertensive disorders, gestational diabetes mellitus, threatened preterm birth, oligohydramnios, premature rupture of membranes, or FGR at the time of infection;
3. A history of antiphospholipid syndrome related clinical events including ≥2 spontaneous miscarriages, Maternal thrombotic events, stillbirth, or early-onset preeclampsia.

### 2.2 Diagnosis and management for SARS-CoV-2 infection in pregnant women

Diagnosis:

1. Having an epidemiological history and clinical manifestations related to viral infection.
2. A positive SARS-CoV-2 nucleic acid or antigen test, or a ≥4-fold increase in the titer of convalescent SARS-CoV-2-specific IgG antibodies compared with the acute phase. The diagnosis is in accordance with the Diagnosis and Treatment Protocol for Novel Coronavirus Infection (Tenth Edition) issued by the National Health Commission of the People’s Republic of China (16, 17).

Management:

1. Adherence to the SARS-CoV-2 infection treatment protocol: Symptomatic treatment was the main approach for infection and post infection recovery. Pregnant women with mild symptoms were isolated at home, with symptomatic interventions including physical cooling, acetaminophen for antipyretic therapy, bromhexine or ambroxol for cough relief, and normal saline nasal irrigation for nasal congestion. For pregnant women with severe or critical SARS-CoV-2 infection, systematic treatment such as antiviral therapy and organ support and protection was administered in the hospital under the guidance of a multidisciplinary team (17, 18).
2. Antenatal care was based on routine prenatal examinations, with close monitoring of maternal and fetal complications, and clinical management was carried out in accordance with the corresponding diagnostic and therapeutic guidelines for complications.

### 2.3 Detection of antiphospholipid antibody profile after SARS-CoV-2 infection

Pregnant women underwent at least one serological aPLs test at 2–4 weeks post infection, Serum levels of anti-cardiolipin antibodies (ACL), anti-beta2-glycoprotein I antibodies (aβ2GPI), and lupus anticoagulant (LAC) were detected. ACL and aβ2GPI were measured using an IgA/G/M multi-subtype screening reagent (EUROIMMUN, Luebeck, Germany) via enzyme linked immunosorbent assay (ELISA). LAC was detected using a modified dilute Russell’s viper venom time (dRVVT) method with the Stago STA Compact Hemostasis System (Diagnostica Stago, Asnières-sur-Seine, France). Normal ranges were: aβ2GPI (0–20 RU/mL), ACL (<10 U/mL), and LAC (0.80-1.20). Values beyond these were considered positive.

### 2.4 Study outcomes

Primary outcomes: The positive rate of aPLs in pregnant women after SARS-CoV-2 infection, and the incidence of placental dysfunction related disorders (including gestational hypertensive disorders, oligohydramnios, or FGR) (13).

Secondary outcomes: The incidence of maternal thrombotic events during pregnancy/puerperium, gestational thrombocytopenia (platelet count <100×10^9^/L), postpartum hemorrhage, as well as neonatal preterm birth, admission to the neonatal intensive care unit (NICU), and birth defects.

### 2.5 Statistical analysis

Data analysis was performed using SPSS 26.0 software. Quantitative data were described as mean ± standard deviation or median (25th percentile, 75th percentile) according to normality test results, and compared using independent samples t test, one-way analysis of variance (ANOVA), or nonparametric tests. Categorical data were presented as number (percentage) and compared using the chi-square test or Fisher’s exact probability test. Univariate logistic regression analysis was used to identify risk factors for adverse maternal and fetal outcomes. A two tailed *P < 0.05* was considered statistically significant.

## 3 Results

### 3.1 General characteristics of the study population and aPLs results after SARS-CoV-2 infection

A total of 80 pregnant women were enrolled in the study, and their baseline characteristics are shown in **Table 1**. The overall positive rate of aPLs in infected pregnant women was 18.75% (15/80). All positive cases were single antibody positivity, either for aβ2GPI (10.00%) or LAC (8.75%); no ACL positivity or co-positivity for ≥2 aPLs was observed.

**Table 1:** General characteristics of the study population.

	Overall population (n=80)	aPLs negative (n=65)	aPLs positivity (n=15)	aβ2GPI positivity (n=8)	LAC positivity (n=7)
Age (year)	33.50 ± 4.39	33.45 ± 4.33	33.73 ± 4.80	34.25 ± 5.39	33.14 ± 4.38
AMA [n (%)]	36 (45.00)	28 (43.08)	8 (53.33)	4 (50.00)	4 (57.14)
Gravidity (times)	2 [1,3]	2 [1,3]	2 [1,3]	2 [1,3]	1 [1,2]
Pluripara [n (%)]	30 (37.50)	25 (38.46)	5 (33.33)	3 (37.50)	2 (28.57)
BMI(Kg/m^2^)	22.39 ± 2.93	22.36 ± 2.94	22.54 ± 2.99	21.92 ± 2.79	23.20 ± 3.29
Obesity [n (%)]	4 (5.00)	3 (4.62)	1(6.67)	1(12.50)	0
Overweight [n (%)]	7 (8.75)	5 (7.69)	2 (13.33)	0	2 (28.57)
ART [n (%)]	7 (8.75)	5 (7.69)	2 (13.33)	0	2 (28.57)
aPLs positivity at different gestational stages (n/%)					
First trimester	17 (21.25)	16/17 (94.12)	1/17 (5.88)	1/17 (5.88)	0
Second trimester	25 (31.25)	21/25 (84.00)	4/25 (16.00)	4/25 (16.00)	0
Third trimester	38 (47.50)	28/38 (73.68)	10/38 (26.32)	3/38 (7.89)	7/38 (18.42)

AMA, advanced maternal age; ART, assisted reproductive technology; aPLs, antiphospholipid antibodies; aβ2GPI, anti-β2-glycoprotein I antibodies; LAC, lupus anticoagulant; BMI, body mass index.

Subgroup analysis of aPLs positivity by gestational trimester at the time of infection showed that the positive rates were 5.88%, 16.00%, and 26.31% in the first, second, and third trimesters, respectively, increasing progressively with gestational advancement. All cases of LAC positivity occurred in pregnant women infected in the third trimester (*P = 0.014*).

### 3.2 aPLs results and maternal and fetal outcomes after SARS-CoV-2 infection

All pregnant women had received at least one dose of inactivated SARS-CoV-2 vaccine before pregnancy. All had mild or asymptomatic SARS-CoV-2 infection and were not hospitalized due to the infection. The mean gestational age at delivery was 39.13 weeks, and there were 80 live births. The incidence of maternal and fetal complications is shown in **Table 2**. No new onset autoimmune diseases were observed in the mothers, and no birth defects were found in the neonates.

**Table 2:** aPLs results and maternal and fetal outcomes after SARS-CoV-2 infection.

	aPLs negative (n=65)	aPLs positivity (n=15)	*P value*	aβ2GPI positivity (n=8)	*P value*	LAC positivity (n=7)	*P value*
Placental dysfunction outcomes	19(29.23)	9(60.00)	*0.036*	4(50.00)	*0.251*	5(71.43)	*0.037*
Gestational Hypertension	8 (12.31)	1 (6.67)	*0.533*	1 (12.50)	*0.988*	0	*0.325*
Preeclampsia	3 (4.62)	2 (13.33)	*0.209*	0	*0.535*	2 (28.57)	*0.018*
Oligohydramnios	5 (7.69)	4 (26.67)	*0.036*	2 (25.00)	*0.117*	2 (28.57)	*0.077*
FGR	5 (7.69)	2 (13.33)	*0.610*	1 (12.50)	*0.515*	1 (14.29)	*0.741*
Gestational diabetes mellitus	9 (13.85)	4 (26.67)	*0.252*	3 (37.50)	*0.119*	1 (14.29)	*>0.999*
Thrombocytopenia	2 (3.08)	0	*>0.999*	0	*0.615*	0	*0.638*
Maternal thrombotic events	1 (1.54)	0	*>0.999*	0	*0.724*	0	*0.741*
PROM	14 (21.54)	2 (13.33)	*0.474*	0	*0.144*	2 (28.57)	*0.671*
Gestational weeks at delivery (Weeks)	39.26 ± 1.14	38.53 ± 1.25	*0.030*	38.27 ± 1.36	*0.026*	38.82 ± 1.16	*0.330*
Postpartum hemorrhage	6 (9.23)	1 (6.67)	*0.751*	0	*0.370*	1 (14.29)	*0.668*
Birth weight (g)	3311 ± 404.6	3192 ± 584.6	*0.350*	3091 ± 431.5	*0.155*	3306 ± 742.5	*0.981*
Premature birth	2 (3.08)	2 (13.33)	*0.156*	1 (12.50)	*0.298*	1 (14.29)	*0.268*
NICU admission	5 (7.69)	1 (6.67)	*>0.999*	0	*>0.999*	1 (14.29)	*0.471*

Data are presented as n (%) or mean ± standard deviation. aPLs, antiphospholipid antibodies; aβ2GPI, anti-β2-glycoprotein I antibodies; LAC, lupus anticoagulant; PROM, premature rupture of fetal membranes; FGR, fetal growth restriction; NICU, neonatal intensive care unit. Two-tailed P <no><</no> 0.05 was considered statistically significant.

Analysis of complications showed that the incidence of placental dysfunction related disorders was significantly higher in the aPLs positive group than in the aPLs negative group (60.00% vs. 29.23%, *P = 0.036*), with an even higher incidence in the LAC positive subgroup (71.43% vs. 29.23%, *P = 0.037*). The incidence of oligohydramnios was significantly higher in the aPLs positive group than in the aPLs negative group (26.67% vs. 7.69%, *P = 0.036*). The incidences of preeclampsia, preterm birth, FGR, and gestational diabetes mellitus showed numerical differences with higher values in the aPLs positive group, with no statistically significant difference Among them, the incidence of preeclampsia in the LAC positive subgroup was significantly higher than that in the aPLs negative group (28.57% vs. 4.62%, *P = 0.018*), and the incidences of oligohydramnios, postpartum hemorrhage, preterm birth, and NICU admission were numerically higher in the LAC-positive subgroup compared with the aPLs negative group (**Table 2**).

### 3.3 Univariate logistic regression analysis of placental dysfunction related disorders in SARS-CoV-2 infected pregnant women

The overall incidence of placental dysfunction related disorders in the study population was 35.00% (28/80). Univariate logistic regression analysis was performed for this disorder considering the basic characteristics of pregnant women, as shown in **Table 3**. The results indicated that aPLs positivity after SARS-CoV-2 infection was a risk factor for placental dysfunction related disorders (OR = 3.632, 95%CI: 1.153 - 12.218, *P* = 0.030), with a higher risk associated with LAC positivity (OR = 6.053, 95%CI: 1.192 - 44.912, *P* = 0.041) (**Table 3**).

**Table 3:** Univariate logistic regression analysis of placental dysfunction related disorders in SARS-CoV-2 infected pregnant women.

	No disorder (n=52)	Disorder (n=28)	Z value	OR (95%CI)	*P* value
General information					
AMA [n (%)]	26 (50.0)	10 (35.7)	-1.219	0.556 (0.210, 1.411)	*0.223*
ART [n (%)]	4 (7.7)	3 (10.7)	0.454	1.440 (0.266, 7.028)	*0.650*
BMI [M(Q1,Q3), kg/m2]	21.8 (20.3, 23.4)	22.4 (20.8, 24.6)	0.898	1.074 (0.918, 1.262)	*0.369*
SARS-CoV-2 infects the gestational stage					
First trimester [n (%)]	14 (26.9)	3 (10.7)	-1.634	0.326 (0.070, 1.123)	0.102
Second trimester [n (%)]	15 (28.8)	10 (35.7)	0.631	1.370 (0.507, 3.641)	0.528
Third trimester [n (%)]	23 (44.2)	15 (53.6)	0.796	1.455 (0.579, 3.703)	0.426
Post-infection aPLs positivity					
aPLs positivity [n (%)]	6 (11.5)	9 (32.1)	2.173	3.632 (1.153, 12.218)	0.030
aβ2GPI positivity [n (%)]	4 (7.7)	4 (14.3)	1.167	2.421 (0.524, 11.228)	0.243
LAC positivity [n (%)]	2 (3.8)	5 (17.9)	2.046	6.053 (1.192, 44.912)	0.041

AMA, advanced maternal age; ART, assisted reproductive technology; BMI, body mass index; aPLs, antiphospholipid antibodies; aβ2GPI, anti-β2-glycoproteinI antibodies; LAC, lupus anticoagulant. OR, odds ratio; CI, confidence interval. Two-tailed P < 0.05 was considered statistically significant.

### 3.4 Clinical characteristics of aPLs positive pregnant women after SARS-CoV-2 infection

The detailed clinical data of the 15 aPLs positive cases are summarized in **Table 4**. None of the patients developed thrombotic events or APS-related clinical manifestations, and no COVID-19 associated complications were observed.

**Table 4:** Clinical characteristics of aPLs positive pregnant women after SARS-CoV-2 infection.

	Age	G/P conception	Infection GA	aPLs post-infection ACL; aβ2GPI; LAC	Follow-up aPLs results	Maternal-fetal adverse outcomes	Delivery details GA, Weight, Length Delivery Mode
1	35	G3P1 Natural	33w	1.8 U/ml 5.41 RU/ml **1.22**	Turned negative at 35 days	FGR Low birth weight infant	38^+3^w, 2160g, 48cm Vaginal delivery
2	37	G1P0 Natural	35w	1.6 U/ml 0.2 RU/ml **1.21**	Not rechecked	Oligohydramnios PPROM, Preterm infant	36^+4^w, 2840g, 47cm Cesarean section
3	40	G4P0 Natural	38w	1.8 U/ml **41.52 RU/ml** 1.15	Not rechecked	GDM	39^+2^w, 2850g, 48cm Cesarean section
4	27	G2P0 Natural	29w	2.1 U/ml 6.8 RU/ml **1.23**	Turned negative at 12 days	Shoulder dystocia Postpartum hemorrhage Macrosomia NICU admission	38^+6^w, 4200g, 51cm Forceps delivery
5	38	G1P0 ART	35w	2 U/ml 9.2 RU/ml **1.24**	Turned negative at 18 days	Preeclampsia PROM	39^+4^w, 3660g, 51cm Vaginal delivery
6	32	G2P1 Natural	23w	1.3 U/ml **56.55 RU/ml** 0.96	aβ2GPI: 25.72 RU/ml at 21 days	Gestational hypertension Thrombocytopenia (PLT: 114×10^9^/L) Preterm labor	36^+6^w, 2980g, 48cm Cesarean section
7	38	G3P1 Natural	36w	1.7 U/ml **95.56 RU/ml** 1.18	Turned negative at 31 days	None	38^+2^w, 3540g, 50cm Cesarean section
8	29	G1P0 ART	33w	1.4 U/ml 15.26 RU/ml **1.38**	Not rechecked	GDM Oligohydramnios	40^+2^w, 3280g, 50cm Vaginal delivery
9	29	G3P0 Natural	7w	1.9 U/ml **21.6 RU/ml** 0.93	Turned negative at 30 days	None	40^+4^w, 3280g, 50cm Vaginal delivery
10	30	G1P0 Natural	37w	2.1 U/ml 2.74 RU/ml **1.22**	Not rechecked	None	39^+1^w, 2880g, 47cm Vaginal delivery
11	41	G1P0 Natural	26w	1.4 U/ml **22.27 RU/ml** 1.2	aβ2GPI: 21.62 RU/ml at 23 days	None	39^+3^w, 3260g, 50cm Cesarean section
12	36	G2P1 Natural	35w	1.3 U/ml 2.49 RU/ml **1.41**	aβ2GPI: 1.37 RU/ml at 14 days	Preeclampsia Macrosomia	38^+6^w, 4125g, 50cm Cesarean section
13	30	G2P1 Natural	22w	2.0 U/ml **46.99 RU/ml** 1.02	aβ2GPI: 22.41 RU/ml at 15 days	Oligohydramnios	37^+2^w, 2680g, 47cm Cesarean section
14	27	G1P0 Natural	16w	1.4 U/ml **32.67 RU/ml** 1.05	Turned negative at 42 days	FGR low birth weight infant	37^+2^w, 2400g, 45cm Cesarean section
15	37	G1P0 Natural	35w	1.1 U/ml **61.92 RU/ml** 1.14	Not rechecked	Oligohydramnios GDM	37^+1^w, 3460g, 50cm Cesarean section

G, gravidity; P, parity; w, week; GA, Gestational Age; aPLs, antiphospholipid antibodies; ACL, anti-cardiolipin antibody; aβ2GPI, anti-beta2 glycoprotein I antibody; LAC, lupus anticoagulant; ART, assisted reproductive technology; NICU, neonatal intensive care unit; PPROM, preterm premature rupture of membrane; FGR, fetal growth restriction; PROM, premature rupture of membrane; GDM, Gestational diabetes mellitus.

Bold values indicate positive antiphospholipid antibody results.

## 4 Discussion

Although relevant studies have noted transient aPLs positivity in individuals after SARS-CoV-2 infection and its correlation with thrombotic risk and pneumonia-related prognosis, research on aPLs status and its impact on pregnancy outcomes in pregnant women with SARS-CoV-2 infection remains scarce. This prospective study observed and analyzed maternal aPLs status and perinatal outcomes in healthy pregnant women infected with SARS-CoV-2 at different gestational stages with a relatively small sample size, and the key findings are discussed in the following aspects.

### 4.1 aPLs status in pregnant women after SARS-CoV-2 infection

Studies have shown that low level aPLs positivity exists physiologically in the general population, causing no significant harm to the body (19, 20). Since the COVID-19 pandemic, numerous studies have revealed a significant increase in the aPLs positive rate after SARS-CoV-2 infection, ranging from 11% to 71% (1, 8, 21). This rate is much higher than the physiological level, and aPLs positivity is closely associated with disease severity and thrombotic diseases.

The variation in the positive rate is thought to be related to differences in patient age, underlying diseases, infection severity, circulating viral strains, vaccination status, and aPLs detection conditions. The largest sample study to date is a meta-analysis reported by Taha et al. in 2025, which included 21 studies with 1159 hospitalized patients with SARS-CoV-2 infection. It showed an overall aPLs positive rate of 46.8% in these patients, with a higher rate in severe cases. Among them, the positive rates of ACL (28.8% vs. 7.10%) and aβ2GPI (12.0% vs. 5.8%) were significantly higher in severe patients (8).

To date, there is only one study on aPLs in pregnant women after SARS-CoV-2 infection. A 2022 investigation by Gozzoli et al. involved 151 hospitalized pregnant patients with SARS-CoV-2 infection, which reported an aPLs positive rate of 11.00% (16/151), including 5 cases of aβ2GPI positivity, 6 cases of ACL positivity, 9 cases of LAC positivity, and 4 cases of co-positivity for two antibodies (15). However, the study population included pregnant women with comorbidities and complications, which cannot represent the aPLs status in healthy pregnant women after SARS-CoV-2 infection.

This study is a small sample study on healthy pregnant women infected with SARS-CoV-2 at different gestational stages. The results showed that the aPLs positive rate in healthy pregnant women after SARS-CoV-2 infection reached 18.75%, which is higher than the baseline physiological level of aPLs in healthy pregnant women (20).

We also found that the aPLs positive rate increased progressively with gestational advancement, which may be related to the changes in maternal immune status during pregnancy. Regarding the duration of aPLs positivity, 10 out of 15 aPLs positive cases underwent re-examination in our study. Among them, 6 cases turned negative (at an interval of 12–42 days), and the remaining 4 cases showed a decrease in antibody titer despite non - negative results (at an interval of 14–21 days). The numerical findings of this study show that aPLs induced by SARS-CoV-2 infection were transiently positive in most cases, which is consistent with the findings reported by Gozzoli et al. In addition, only single antibody positivity for aβ2GPI (10.00%) or LAC (8.75%) was observed in our study, with no co-positivity for multiple antibodies.

### 4.2 Analysis of the characteristics of aPLs positivity and maternal and fetal outcomes after SARS-CoV-2 infection

There is currently no clear conclusion on whether aPLs positivity after SARS-CoV-2 infection directly affects maternal outcomes (10, 11). Although most reports suggest that SARS-CoV-2 infection increases the risk of adverse maternal and fetal outcomes, and an elevated incidence of placental function related pregnancy disorders such as gestational hypertensive disorders and FGR has been observed in infected pregnant women, the specific mechanism remains unclear.

This study found that the incidence of gestational complications was increased in the aPLs positive group, especially in the LAC positive subgroup, particularly for placental dysfunction related adverse outcomes including gestational hypertensive disorders, oligohydramnios, and FGR. The incidences of preeclampsia, preterm birth, FGR, and gestational diabetes mellitus showed numerical differences with higher values in the aPLs positive group, with no statistically significant difference.

The underlying mechanisms may be explained as follows: On the one hand, drawing on studies on antiphospholipid syndrome, aPLs can induce pathological pregnancy through multiple pathways, with aβ2GPI and LAC specifically involved in placental dysfunction. Previous studies have suggested that aPLs elevation induced by SARS-CoV-2 infection can mediate pathological hypercoagulability and exacerbate tissue damage (3, 22). Among aPLs, LAC has been proven to be the antibody most closely associated with thrombotic events (14, 23), and studies on pathological pregnancy in antiphospholipid syndrome have confirmed that LAC is an independent risk factor for adverse pregnancy outcomes among aPLs (14). In addition, the significant increase in the aPLs positive rate after SARS-CoV-2 infection has been widely observed in the general population, suggesting that SARS-CoV-2 may mediate placental dysfunction through infection induced aPLs positivity, thereby leading to adverse pregnancy outcomes.

On the other hand, aPLs status after infection is associated with the maternal systemic immune status (24), and aPLs positivity also indicates excessive activation of the maternal immune system induced by infection; the systemic inflammatory response is also a fundamental factor for adverse maternal and fetal outcomes.

Stratified analysis by gestational trimester at the time of infection in this study showed that the aPLs positive rate increased progressively with gestational advancement (5.88%, 16.00%, 26.31%), with the most obvious trend observed for LAC. All cases of LAC positivity in this study occurred in pregnant women infected in the third trimester. Infection-induced LAC positivity overlaps with the physiological hypercoagulability inherent in the third trimester of pregnancy (25). Numerical associations are observed between this overlap and elevated maternal-fetal adverse outcome rates.

### 4.3 Clinical management of pregnant women with SARS-CoV-2 infection

With the evolutionary trend of most SARS-CoV-2 variants towards enhanced transmissibility and attenuated pathogenicity, since the implementation of normalized COVID-19 control measures, most infections have been mild or asymptomatic, and symptomatic treatment is the main approach for viral infection. However, the management of pregnant infected individuals still warrants in-depth discussion.

Based on the results of this study, we believe that antenatal management should emphasize the value of aPLs screening in pregnant women with SARS-CoV-2 infection, especially those infected in the second or third trimester, as well as pregnant women with high-risk factors for gestational hypertensive disorders and other conditions that affect placental function. After infection, through aPLs screening, enhanced monitoring of placental function should be implemented for aPLs positive pregnant women to identify and intervene in adverse maternal and fetal outcomes in a timely manner.

In addition, when clinical manifestations of placental dysfunction related disorders such as FGR, preeclampsia, and oligohydramnios occur, if the pregnant woman has a recent history of SARS-CoV-2 infection, active serological aPLs screening should be performed to identify potential high-risk populations.

This is an observational study, and all complications identified during maternal and fetal monitoring were managed in accordance with relevant guidelines and diagnostic and therapeutic protocols, with no specific interventions based on aPLs test results. Although the overall maternal and fetal outcomes were favorable, the incidence of placental dysfunction related adverse outcomes was still higher in aPLs positive cases.

For aPLs positive pregnant women after SARS-CoV-2 infection, it is essential to be vigilant about infection deterioration through vital signs and inflammatory indicators, and to closely monitor coagulation function to guard against thrombotic events. From an obstetric perspective, surveillance of placental function and maternal-fetal status for this population should be further enhanced. Therefore, to further improve maternal and fetal outcomes, the value of prophylactic intervention for pregnant women with positive aPLs on screening remains to be explored. In particular, whether low-molecular-weight heparin should be administered for prophylactic anticoagulation in LAC-positive cases to mitigate pathological hypercoagulability and reduce the incidence of placental origin disorders requires confirmation in larger-scale cohort studies.

### 4.4 Study limitations

This is a single center prospective cohort study. All the enrolled cases are from the prenatal care population of our hospital, ensuring detailed and accurate data. Although the study was conducted during a brief period of normalized COVID-19 control in China, the sample size was limited. In addition, for pregnant women without comorbidities, there was a lack of baseline aPLs data before SARS-CoV-2 infection and continuous dynamic antibody monitoring. This may have limited the in depth analysis of the dynamic changes and clinical significance of aPLs.

## 5 Conclusions

This study found that healthy pregnant women without comorbidities or complications may develop transiently increased aPLs positivity after SARS-CoV-2 infection, and aPLs positivity after infection is a risk factor for placental dysfunction related adverse perinatal outcomes. These findings suggest that in clinical practice, close attention should be paid to the history of SARS-CoV-2 infection during pregnancy, especially infection in the third trimester. Moreover, importance should be attached to aPLs screening in infected pregnant women. For aPLs positive cases after SARS-CoV-2 infection, enhanced maternal and fetal monitoring, vigilant surveillance of infection status and coagulation function, and follow-up are necessary.

## Data Availability

The original contributions presented in the study are included in the article/supplementary material. Further inquiries can be directed to the corresponding authors.
